# Characterization of highly pathogenic clade 2.3.4.4b H5N1 mink influenza viruses

**DOI:** 10.1016/j.ebiom.2023.104827

**Published:** 2023-10-07

**Authors:** Tadashi Maemura, Lizheng Guan, Chunyang Gu, Amie Eisfeld, Asim Biswas, Peter Halfmann, Gabriele Neumann, Yoshihiro Kawaoka

**Affiliations:** a Department of Pathobiological Sciences, Influenza Research Institute, School of Veterinary Medicine, University of Wisconsin-Madison, 575 Science Drive, Madison, WI 53711, USA; b Division of Virology, Department of Microbiology and Immunology, International Research Center for Infectious Diseases, The Institute of Medical Science, University of Tokyo, Tokyo 108-8639, Japan; c Research Center for Global Viral Diseases, National Center for Global Health and Medicine, Tokyo 162-8655, Japan; d The University of Tokyo, Pandemic Preparedness, Infection and Advanced Research (UTOPIA) Center, Tokyo 108-8639, Japan

Highly pathogenic avian H5 influenza viruses (HPAI) have caused more than 957 human infections, but do not transmit via respiratory droplets among mammals, although genetically modified HPAI H5 viruses can acquire this ability.^1^^,^^2^ HPAI H5N1 viruses of clade 2.3.4.4b emerged in Europe in late 2020 and have caused major outbreaks in multiple continents. Mass mortality events of 2.3.4.4b clade viruses in mink^3^ and sea mammals^4^ suggest high infectivity and pathogenicity among mammals. A recent study evaluated the pathogenicity and transmissibility of several clade 2.3.4.4b H5N1 viruses isolated in Canada.^5^ One of the H5N1 viruses tested (A/Red Tailed Hawk/ON/FAV-0473-4/2022) transmitted to five of six contact ferrets in the same cage, but transmission of infectious virus via respiratory droplets was not detected. For the clade 2.3.4.4b viruses that caused an outbreak on a mink farm in Spain, pathogenicity and respiratory droplet transmission among mammals (a prerequisite for causing a pandemic) are unknown. These viruses did not acquire the mammalian-adapting PB2-E627K substitution,^3^ but encode PB2-271A, which enhances viral polymerase activity in mammalian cells,^6^ suggesting high pathogenicity in mammals. Here, we tested the pathogenicity and potential of the mink viruses to transmit via respiratory droplets among mammals.

We generated two clade 2.3.4.4b mink viruses isolated from the outbreak in Spain (A/mink/Spain/22VIR12774-13_3869-2/2022 (mink 3869-2), A/mink/Spain/22VIR12774-14_3869-3/2022 (mink 3869-3)) which differ by several amino acids (Appendix pp. 5) and intranasally inoculated mice with each virus. Both viruses were highly virulent with similar mouse lethal dose 50 values (MLD_50_s) of 48.1 and 30.0 plaque-forming units (PFU), respectively; however, their MLD_50_s were higher than that of highly virulent A/Vietnam/1203/2004 (VN1203) virus (MLD_50_: 2.2 PFU) (Appendix pp. 6). In mice infected with 10^3^ PFU of the mink viruses, we detected high virus titers in the respiratory organs as well as systemic spread in the brain, spleen, liver, colon, kidney, and heart (Appendix pp. 7). However, the mink virus titers in most organs were lower than the VN1203 titers.

To test the respiratory droplet transmissibility of the mink viruses, we intranasally inoculated groups of three ferrets with 10^6^ PFU of each virus. One day later, naïve ferrets were placed into neighboring cages that prevented direct contact between the inoculated and exposed animals but allowed virus respiratory droplet transmission through the air. Nasal swab samples collected after inoculation or exposure revealed robust virus titers in infected, but not exposed animals (Fig. 1). Moreover, the exposed animals did not seroconvert (Appendix pp. 8). In contrast, a human seasonal influenza virus known to transmit efficiently via respiratory droplets in ferrets (A/Isumi/UT-KK001-01/2018, H1N1pdm) transmitted in all three pairs of ferrets (Fig. 1). In addition, we found that the sera collected on Day 10 post-infection or Day 9 post-exposure (before the ferrets were euthanized) showed high hemagglutination inhibition titers against the homologous virus (Appendix pp. 8), validating our experimental setup.

**Fig. 1:**
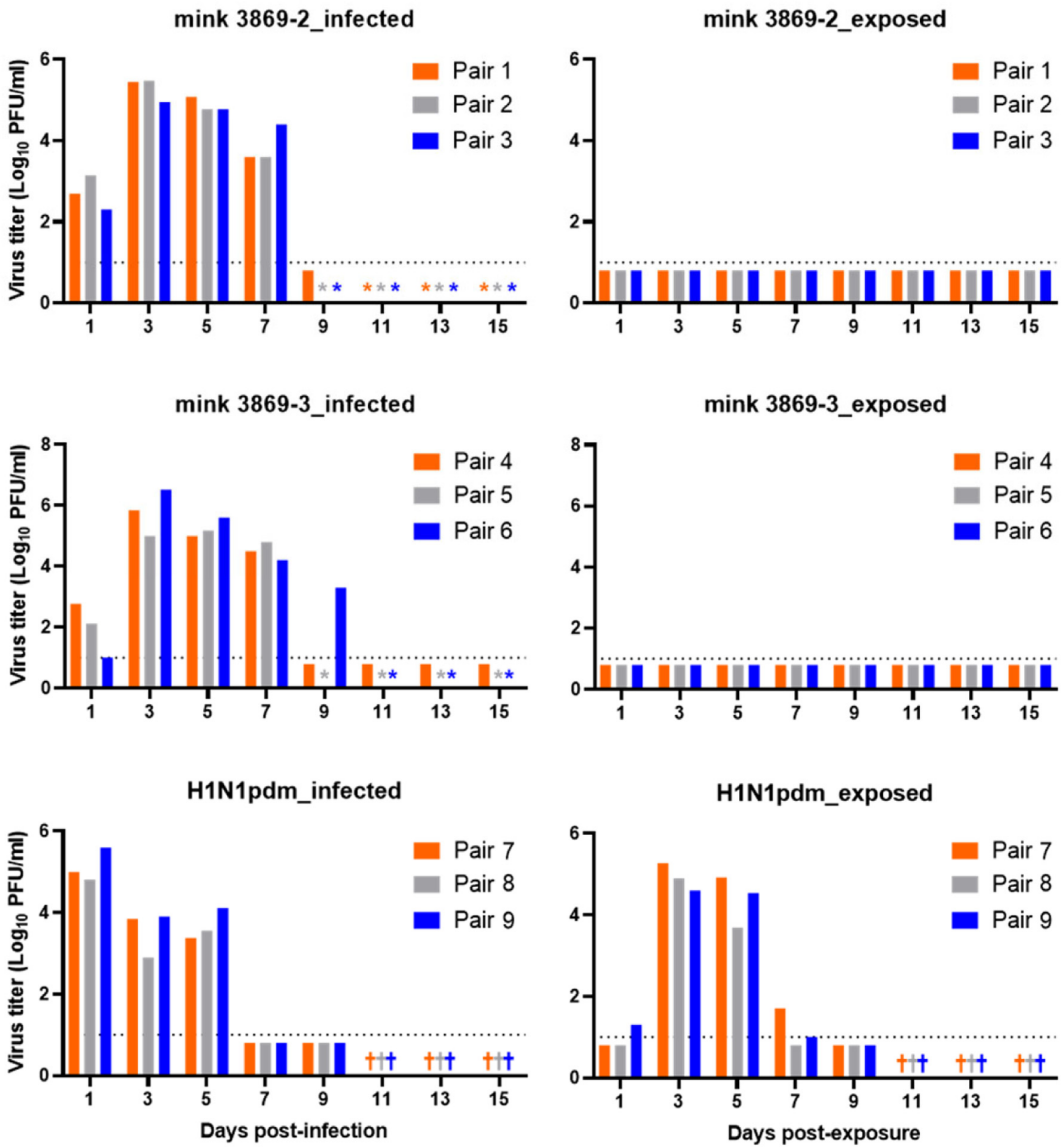
**Virulence and transmissibility of clade 2.3.4.4b mink H5N1 viruses in ferrets.** Ferrets (three per group) were inoculated with 10^6^ plaque-forming units (PFU) of A/mink/Spain/22VIR12774-13_3869-2/2022 (H5N1, mink 3869-2), A/mink/Spain/22VIR12774-14_3869-3/2022 (H5N1, mink 3869-3), or A/Isumi/UT-KK001-01/2018 (H1N1, H1N1pdm) virus. One day later, naïve animals were placed into cages that allowed air flow, but no direct contact between the infected and exposed animals. Nasal swab samples were collected at the indicated timepoints and virus titers were determined by performing plaque assays with MDCK cells. ∗Ferrets met euthanasia criteria. ^†^Ferrets were euthanized on Day 10 post-infection or Day 9 post-exposure, respectively, because the virus infection had cleared. The dotted line indicates the detection limit (1.0 log_10_ PFU/ml).

All ferrets infected with mink 3869-2 virus and two of the three ferrets infected with mink 3869-3 virus experienced substantial weight loss (ranging from 17.0% to 37.9%; see Appendix pp. 9) or were unable to remain upright and had to be euthanized on Days 8–10 post-infection. Virus titrations of dissected organs of these ferrets showed systemic virus replication (Appendix pp. 9), consistent with the systemic virus spread in infected ferrets euthanized on Days 3 and 6 post-infection for virus titrations (Appendix pp. 10). Although the mink virus titers in the non-respiratory organs including brain, spleen, heart, and liver were lower than those of VN1203 in ferrets, mink 3869-3 virus showed higher virus titers in the fecal samples. In the colon, robust virus titers were detected on Day 6 post-infection for ferrets infected with VN1203 or mink viruses; no statistically significant differences were detected among the virus titers. These data suggest replication of mink viruses in the gastrointestinal tract.

Collectively, our data demonstrate that two different clade 2.3.4.4b mink viruses are highly virulent in mice and ferrets but do not transmit to exposed ferrets through respiratory droplets.

## Contributors

TM, PH, GN, and YK designed the study. TM, LG, CG, AE, and AB performed the mouse and ferret studies. TM, GN, and YK analyzed the data and wrote the manuscript. TM, LG, and CG contributed equally. TM, LG, and CG accessed and verified the underlying data reported in the manuscript. All authors read and approved the final version of the manuscript.

## Data sharing statement

Data supporting the results of this study are included in the paper and/or supplementary materials. Additional data related to this paper can be requested from the authors.

## Declaration of interests

GN and YK are co-founders of FluGen. YK is funded by a Research Program on Emerging and Reemerging Infectious Diseases (JP21fk0108552 and JP21fk0108615), by a Project Promoting Support for Drug Discovery (JP21nf0101632), by the Japan Program for Infectious Diseases Research and Infrastructure (JP23wm0125002), and by the Japan Initiative for World-leading Vaccine Research and Development Centers (JP233fa627001) from the Japan Agency for Medical Research and Development. YK has received unrelated funding support from Daiichi Sankyo Pharmaceutical, Fuji Film (Toyama Chemical), Tauns Laboratories, Shionogi, Otsuka Pharmaceutical, KM Biologics, Kyoritsu Seiyaku, Shinya Corporation, and Fuji Rebio.

All other authors declare no competing interests.
