# Supplementary material for: Characterization of highly pathogenic clade 2.3.4.4b H5N1 mink influenza viruses
Source: eBioMedicine. 2023 Oct 7;97:104827. doi: 10.1016/j.ebiom.2023.104827 (PMC10579283; doi:10.1016/j.ebiom.2023.104827)
Supplement: Appendix [file mmc1.pdf]

Supplementary appendix

Supplement to: Maemura T, Guan L, Gu C, et al. Characterization of highly pathogenic clade 2.3.4.4b H5N1 mink influenza viruses.

Supplementary Appendix

Supplementary Materials and Methods

Plasmid construction and reverse genetics. The viral cDNAs of A/mink/Spain/22VIR12774-14_3869-3/2022 (GISAID EPI_ISL-16507096) were generated by PCR-amplification of gBlock gene fragments (IDT DNA) designed based on the published sequence, and then cloned into RNA polymerase I-based plasmid vectors for reverse genetics [1]. A/mink/Spain/22VIR12774-13_3869-2/2022 (GISAID EPI_ISL_16507095) differs from A/mink/Spain/22VIR12774-14_3869-3/2022 by amino acid changes in the PB2, PA, NA, and NS1 proteins (Supplementary Table S1). These amino acid changes were introduced into A/mink/Spain/22VIR12774-14_3869-3/2022 by site-directed mutagenesis (PrimeSTAR® Mutagenesis Basal Kit, Takara Bio Inc., Shiga, Japan), thereby creating cDNAs for the generation of A/mink/Spain/22VIR12774-13_3869-2/2022.

Viruses were generated by transfecting human embryonic kidney (293T) cells with eight RNA polymerase I plasmids encoding the viral RNAs of A/mink/Spain/22VIR12774-14_3869-3/2022 (mink 3869-3) or A/mink/Spain/22VIR12774-13_3869-2/2022 (mink 3869-2), respectively, and protein expression plasmids encoding influenza virus polymerase and NP proteins, using established protocols [1]. Virus-containing supernatant was collected 48 h later and amplified and titrated in Madin-Darby canine kidney (MDCK) cells. Prior to use of the viruses in cell culture and animal studies, the sequences of both viral genomes were confirmed by Sanger sequencing. A/Vietnam/1203/2004 (VN1203; H5N1) [2] and A/Isumi/UT-KK001-1/2018 (H1N1pdm) (provided by the University of Tokyo) served as control viruses because of their high pathogenicity in mice and ferrets and their transmissibility in ferrets, respectively.

Cells. Human embryonic kidney (293T) cells were maintained in DMEM supplemented with 10% fetal bovine serum (FBS). MDCK cells were grown in MEM containing 5% newborn calf serum (NCS). All cells were incubated at 5% CO_2 and 37 °C.

Mouse studies. To determine the mouse lethal dose 50, six-week-old female BALB/c mice (Jackson Laboratories, Bar Harbor, ME, USA; four mice per group) were intranasally inoculated with 10^0, 10^1, 10^2, 10^3, 10^4, 10^5, or 10^6 plaque-forming units (PFU) (in 50 µl) of mink 3869-2, mink 3869-3, or VN1203 under anesthesia with isoflurane. Body weight change and survival were monitored daily for 14 days. Infected mice were euthanized if they lost more than 35% of their initial body weight. Lethal dose 50 values were calculated according to the method of Reed and Muench [3]. For virological examinations, 10 mice per group were intranasally inoculated with 10^3

PFU of the viruses and five mice per group were euthanized at 3- and 6-days post-infection. Virus titers in the lung, nasal turbinate, trachea, heart, brain, liver, spleen, kidney, and colon were determined by performing plaque assays in MDCK cells.

Ferret studies. For this study, 5–7-month-old female ferrets (Triple F Farms) that were serologically negative by the hemagglutinin inhibition assay for currently circulating human influenza viruses were used. For pathogenicity studies, eight animals per group were intramuscularly anaesthetized and intranasally inoculated with 10^6 PFU (250 µl per nostril) of virus. On Days 3 and 6 post-infection, four ferrets per group were euthanized. Virus titers in several organs were determined by performing plaque assays in MDCK cells.

For transmission studies in ferrets, animals were infected as described above. Twenty-four hours later, naïve ferrets were each placed in a cage adjacent to an inoculated ferret. The transmission cages used in this study prevent direct contact between animals but allow spread of influenza virus through the air. To assess virus replication in inoculated and exposed ferrets, nasal swab samples were collected on Day 1 after inoculation or exposure, respectively, and then every other day, followed by virus titration in MDCK cells. Virus titers in the organs of ferrets that succumbed to their infection during the experiment were determined by performing plaque assays in MDCK cells. Sera were collected at the end points.

Hemagglutination inhibition (HI) assay. Ferret sera were treated with receptor-destroying enzyme (RDE II, Denka Seiken) at 37 °C for 20 h, followed by RDE inactivation at 56 °C for 50 min and absorption with turkey red blood cells (TRBCs) at room temperature for 1 h. The treated sera (25 µl) were serially two-fold diluted with PBS in 96-well V-bottom microtiter plates and mixed with the amount of the virus equivalent to four hemagglutination units (25 µl), followed by incubation at room temperature for 30 min. After addition of 50 µl of 0.5% TRBCs, the mixtures were gently mixed and incubated at room temperature for 45 min. HI titers are expressed as the inverse of the highest serum dilution that inhibited hemagglutination.

Statistics

GraphPad Prism software was used to analyze the data. Virus titers in the organ tissues on each day are presented as the mean ± standard deviation (mice: n = 5; ferrets: n = 4); statistical significance was determined by using the Mann-Whitney test to evaluate the difference between the mink viruses and VN1203 (p value < 0.05; Supplementary Figures S2 and S3).

Ethics. All studies were reviewed and approved by the University of Wisconsin-Madison's Institutional Biosafety Committee (IBC). This manuscript was reviewed by the University of Wisconsin-Madison Dual Use Research of Concern (DURC) Subcommittee. This review was conducted in accordance with the United States Government September 2014 DURC Policy. The DURC Subcommittee concluded that the studies described herein do not meet the criteria of Dual Use Research of Concern (DURC). Animal studies were performed in accordance with the Animal Care and Use Committee guidelines of the University of Wisconsin-Madison (protocol #V6426).

Role of funders. The funders of this study had no role in the study design, data collection, analysis, interpretation, or the writing of the manuscript.

Acknowledgement

We thank Susan Watson for scientific editing.

Supplementary Tables and Figures

Supplementary Table S1. Comparison of the amino acid sequences of the clade 2.3.4.4b mink viruses characterized in this study*

Virus	Collection date	PB2		PB1	PA		NA	NS1			NS2 (NEP)
		138	598	480	349	716	395	73	155	C-terminus	74
A/mink/Spain/22VIR12774-13_3869-2/2022**	10/26/2022	R	T	X**	E	N	A	P	A	WRNEMVD	G
A/mink/Spain/22VIR12774-14_3869-3/2022**	10/26/2022	Q	I	K	X**	K	T	S	V	-------	E

*Shown are only proteins with amino acid differences between the two mink H5N1 virus isolates.
**The published sequences list an 'X'. Based on the comparison with other clade 2.3.4.4b mink viruses, the consensus amino acids (i.e., PB1-480K and PA-349E) were used here.

Supplementary Figure S1

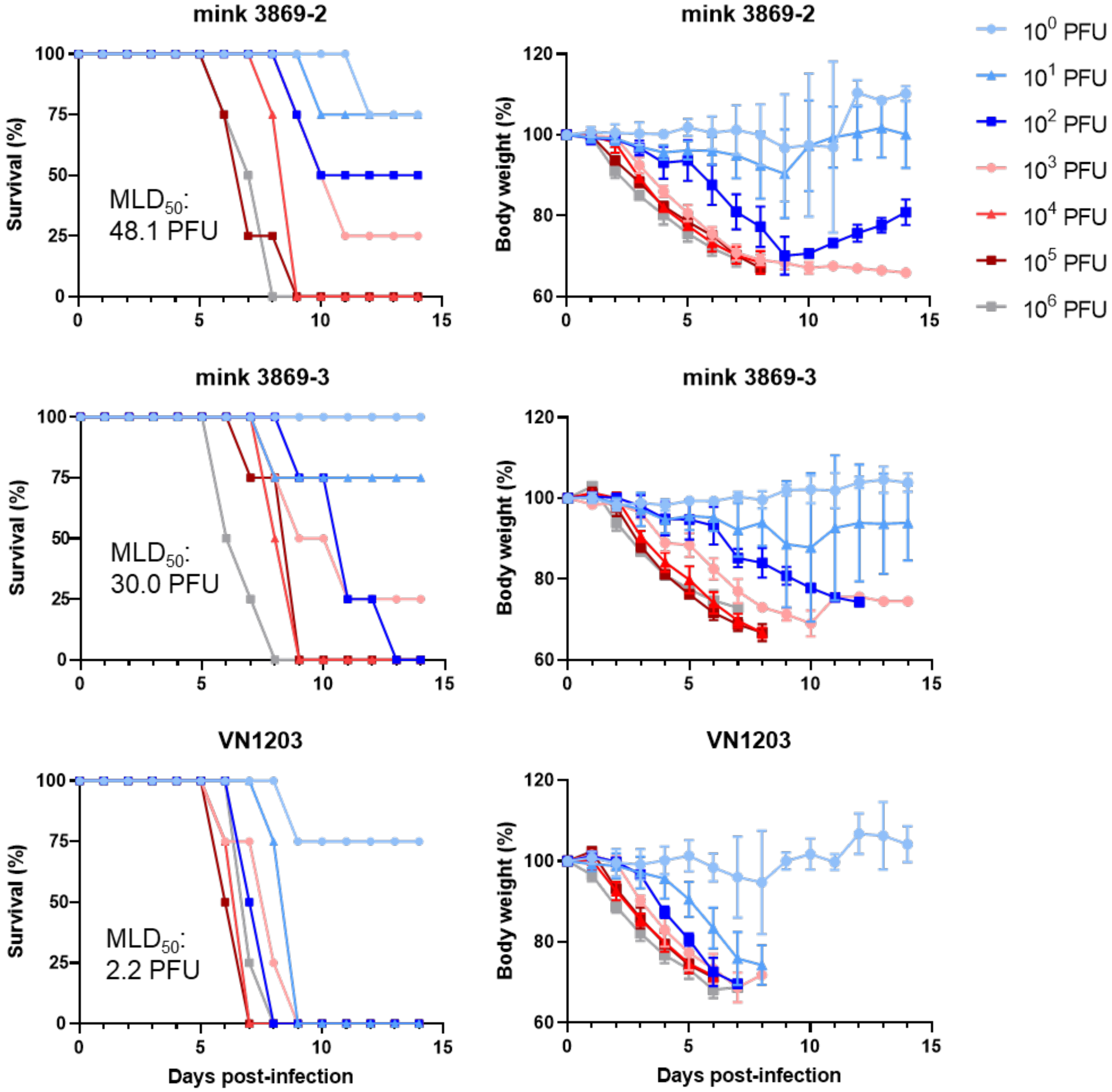

Supplementary Figure S1 legend

Virulence of clade 2.3.4.4b mink H5N1 viruses in mice. Mice (four per group) were inoculated with the indicated dose of A/mink/Spain/22VIR12774-13_3869-2/2022 (H5N1, mink 3869-2), A/mink/Spain/22VIR12774-14_3869-3/2022 (H5N1, mink 3869-3), or A/Vietnam/1203/2004 (H5N1, VN1203) virus. Body weight changes and survival were monitored daily. Error bars show the mean ± standard deviation.

Supplementary Figure S2

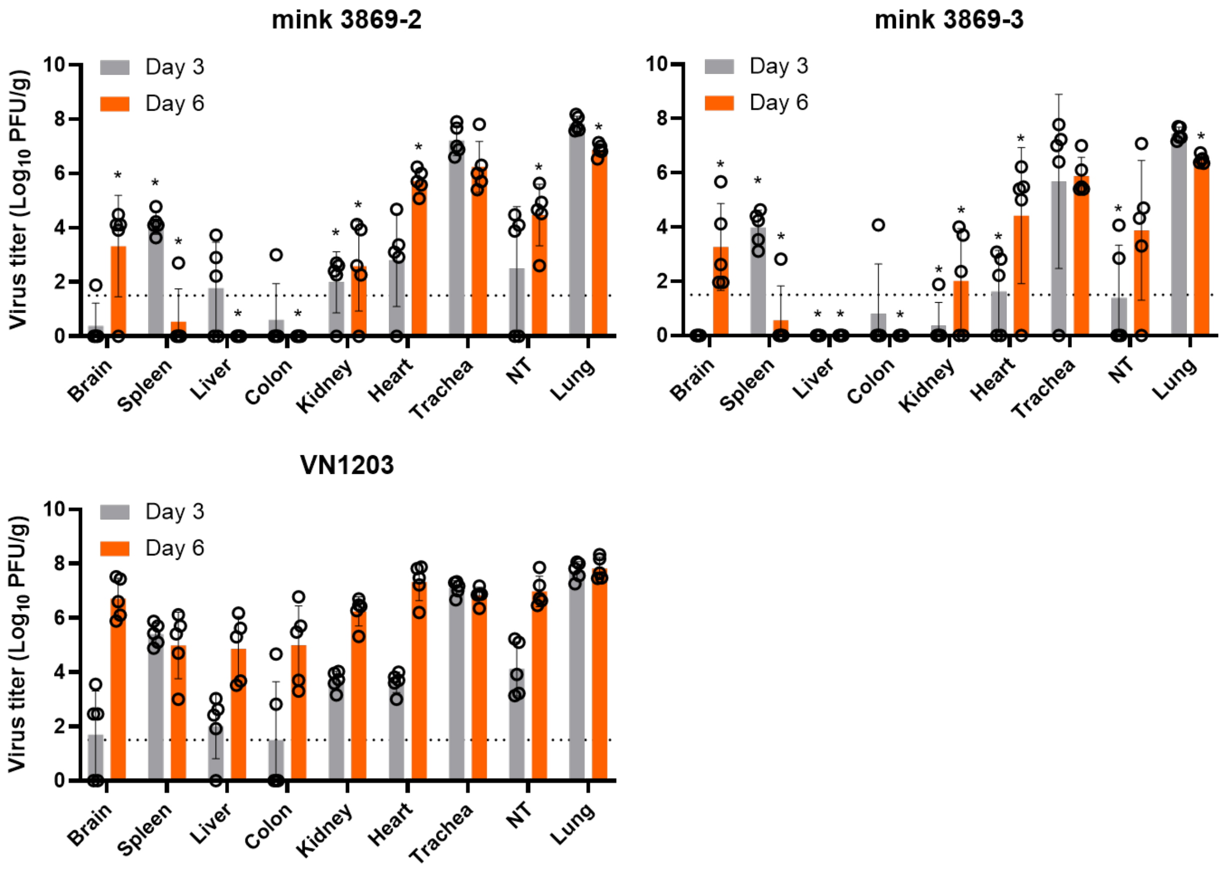

Supplementary Figure S2 legend

Pathogenicity of clade 2.3.4.4b mink H5N1 viruses in mice. Mice (ten per group) were inoculated with 10^3 plaque-forming units (PFU) of A/mink/Spain/22VIR12774-13_3869-2/2022 (H5N1, mink 3869-2), A/mink/Spain/22VIR12774-14_3869-3/2022 (H5N1, mink 3869-3), or A/Vietnam/1203/2004 (H5N1, VN1203) virus. On Days 3 and 6 post-infection, five animals in each group were euthanized and virus titers in the indicated mouse organs were determined by performing plaque assays in MDCK cells. NT, nasal turbinate. Bars indicate mean values and each point indicates data from an individual animal. Error bars show the mean ± standard deviation. The dotted line indicates the detection limit (1.5 $\log_{10}$ PFU/g). Statistical analysis was performed by using the Mann-Whitney test. An asterisk (*) indicates a significant difference between the respective mink virus and VN1203 ($p < 0.05$).

Supplementary Table S2. HI titers of infected and exposed ferrets against homologous virus

Virus	Group	Pair	Ferret ID	Pre-Infection	Post-infection/-exposure*
mink 3869-2	Infected	P1	T1242	<10	<10 (euthanized on Day 8)**
		P2	T1237	<10	<10 (euthanized on Day 8)**
		P3	T1244	<10	<10 (euthanized on Day 10)**
	Exposed	P1	T1243	<10	<10
		P2	T1238	<10	<10
		P3	T1245	<10	<10
mink 3869-3	Infected	P1	T1240	<10	80
		P2	T1074	<10	20 (euthanized on Day 8)**
		P3	T1223	<10	20 (euthanized on Day 9)**
	Exposed	P1	T1241	<10	<10
		P2	T1075	<10	<10
		P3	T1224	<10	<10
H1N1pdm	Infected	P1	T1233	<10	5120 (euthanized on Day 10)***
		P2	T1235	<10	10240 (euthanized on Day 10)***
		P3	T301	<10	10240 (euthanized on Day 10)***
	Exposed	P1	T1234	<10	10240 (euthanized on Day 9)***
		P2	T1236	<10	10240 (euthanized on Day 9)***
		P3	T1222	<10	10240 (euthanized on Day 9)***

*Sera were collected on Day 21 post-infection or -exposure, respectively, unless otherwise indicated.
**Sera were collected because the ferrets met the euthanasia criteria.
***Sera were collected because virus had been cleared.

Supplementary Table S3. Body weight loss and virus titers in ferrets that had to be euthanized or succumbed to clade 2.3.4.4b H5N1 mink virus infection on days 8-10 post-infection*

Virus	Day of death	Animal ID	Body weight loss (%)	Brain	Spleen	Liver	Colon	Feces	Kidney	Heart	Trachea	Nasal Turbinate	Lung
mink 3869-2	8	Infected_Pair 2 (T1237)	37.9	-	-	-	-	3.15	-	-	-	3.46	3.12
mink 3869-2	8	Infected_Pair 3 (T1244)	25.8**	7.48	-	2.35	-	-	-	-	1.62	6.00	2.98
mink 3869-3	8	Infected_Pair 2 (T1074)	17.0**	6.96	-	6.62	3.15	-	2.37	2.37	6.73	7.96	6.35
mink 3869-3	9	Infected_Pair 3 (T1223)	31.2**	6.14	2.48	5.51	-	2.89	-	-	6.35	8.18	6.74
mink 3869-2	10	Infected_Pair 1 (T1242)	33.5**	4.82	-	2.94	-	-	2.15	-	-	5.84	-

*Titers shown are $\log_{10}$ PFU/g.

**These ferrets were euthanized due to their inability to remain upright, in accordance with the Animal Care and Use Committee guidelines of the University of Wisconsin-Madison (protocol #V6426).

Supplementary Figure S3

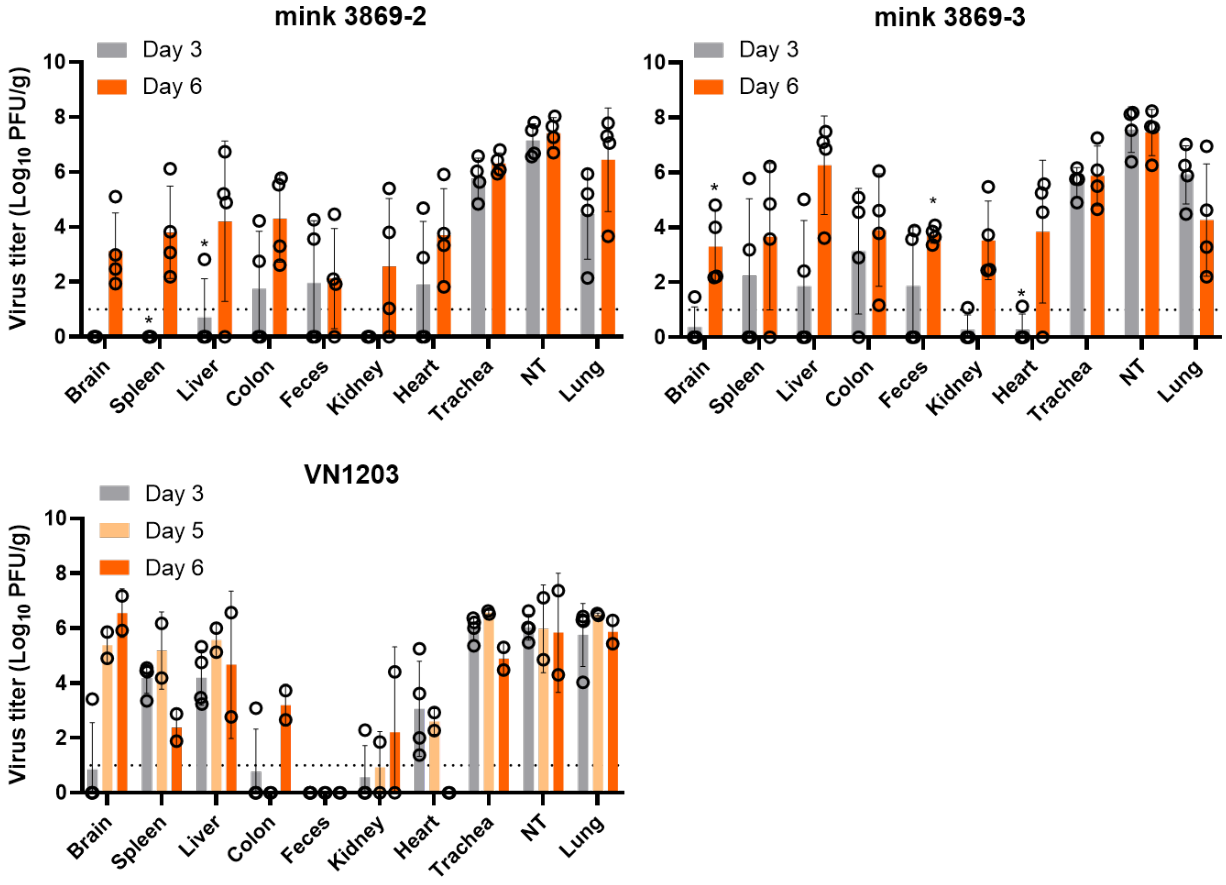

Supplementary Figure S3 legend

Pathogenicity of clade 2.3.4.4b mink H5N1 viruses in ferrets. Ferrets (eight per group) were inoculated with 10^6 plaque-forming units (PFU) of A/mink/Spain/22VIR12774-13_3869-2/2022 (H5N1, mink 3869-2), A/mink/Spain/22VIR12774-14_3869-3/2022 (H5N1, mink 3869-3), or A/Vietnam/1203/2004 (H5N1, VN1203) virus. On Days 3 and 6 post-infection, four animal from each group were euthanized and virus titers in the indicated ferret organs were determined by performing plaque assays in MDCK cells. Two of the ferrets infected with VN1203 died on Day 5 post-infection, whereas the other two were euthanized on Day 6 post-infection. NT, nasal turbinate. Bars indicate mean values and each point indicates data from an individual animal. Error bars show the mean ± standard deviation. The dotted line indicates the detection limit (1.0 $\log_{10}$ PFU/g). Statistical analysis was performed by using the Mann-Whitney test. An asterisk (*) indicates a significant difference between the respective mink virus and VN1203 (p < 0.05). For the statistical analyses, the VN1203 samples on Day 5 were combined with those from Day 6 (n = 4).

References

1. Neumann G, Watanabe T, Ito H, et al. Generation of influenza A viruses entirely from cloned cDNAs. *Proc Natl Acad Sci U S A* 1999; **96**(16): 9345-50.
2. Hatta M, Hatta Y, Kim JH, et al. Growth of H5N1 influenza A viruses in the upper respiratory tracts of mice. *PLoS Pathog* 2007; **3**(10): 1374-9.
3. REED LJ, MUENCH H. A SIMPLE METHOD OF ESTIMATING FIFTY PER CENT ENDPOINTS12. *American Journal of Epidemiology* 1938; **27**(3): 493-7.
